# An integrated in utero MR method for assessing structural brain abnormalities and measuring intracranial volumes in fetuses with congenital heart disease: results of a prospective case-control feasibility study

**DOI:** 10.1007/s00234-019-02184-2

**Published:** 2019-02-22

**Authors:** Paul D. Griffiths, Hatem A. Mousa, Chloe Finney, Cara Mooney, Laura Mandefield, Timothy J. A. Chico, Deborah Jarvis

**Affiliations:** Academic Unit of Radiology, University of Sheffield, Floor C, Royal Hallamshire Hospital, Glossop Road, Sheffield, S10 2JF UK

**Keywords:** Fetus, Magnetic resonance imaging, Congenital heart disease, Brain abnormality

## Abstract

**Purpose:**

To refine methods that assess structural brain abnormalities and calculate intracranial volumes in fetuses with congenital heart diseases (CHD) using in utero MR (iuMR) imaging. Our secondary objective was to assess the prevalence of brain abnormalities in this high-risk cohort and compare the brain volumes with normative values.

**Methods:**

We performed iuMR on 16 pregnant women carrying a fetus with CHD and gestational age ≥ 28-week gestation and no brain abnormality on ultrasonography. All cases had fetal echocardiography by a pediatric cardiologist. Structural brain abnormalities on iuMR were recorded. Intracranial volumes were made from 3D FIESTA acquisitions following manual segmentation and the use of 3D Slicer software and were compared with normal fetuses. *Z* scores were calculated, and regression analyses were performed to look for differences between the normal and CHD fetuses.

**Results:**

Successful 2D and 3D volume imaging was obtained in all 16 cases within a 30-min scan. Despite normal ultrasonography, 5/16 fetuses (31%) had structural brain abnormalities detected by iuMR (3 with ventriculomegaly, 2 with vermian hypoplasia). Brain volume, extra-axial volume, and total intracranial volume were statistically significantly reduced, while ventricular volumes were increased in the CHD cohort.

**Conclusion:**

We have shown that it is possible to perform detailed 2D and 3D studies using iuMR that allow thorough investigation of all intracranial compartments in fetuses with CHD in a clinically appropriate scan time. Those fetuses have a high risk of structural brain abnormalities and smaller brain volumes even when brain ultrasonography is normal.

## Introduction

Congenital heart disease (CHD) is the commonest developmental abnormality in live births (prevalence 8/1000) and the likeliest to cause death [1]. Improved treatment, particularly surgical repair, has greatly improved long-term survival resulting in an increased prevalence of CHD in adults to approximately 4/1000 [2]. Up to 50% of children with severe CHD have some degree of neuro-developmental difficulty [3–8], and the American Heart Association recommends serial neuro-developmental screening and monitoring for those at highest risk [9]. The etiology of the neuro-developmental problems is complex and multifactorial; undoubtedly, complications from surgery [9–13] contribute but known pre- and peri-operative risk factors are thought to account for around 30% of poor neuro-developmental outcomes.

**Fig. 1:**
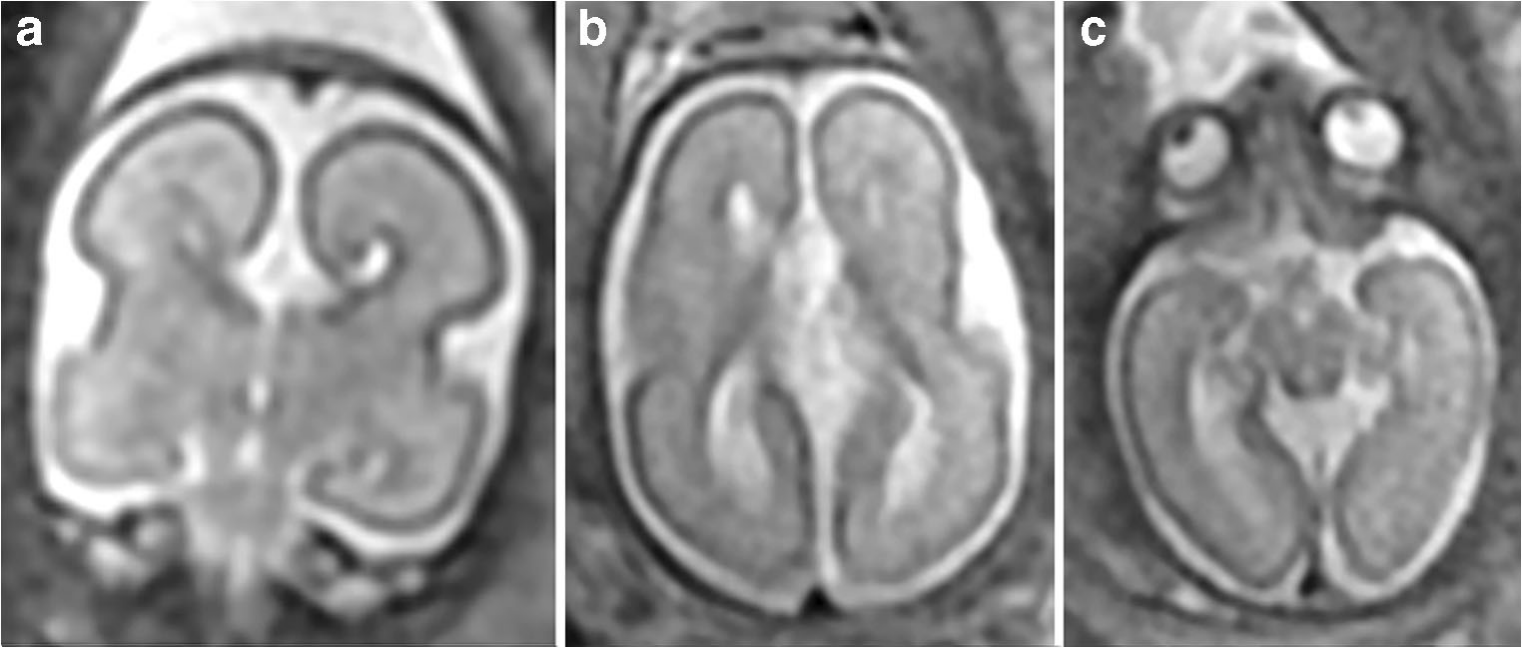
A fetus with known atrioventricular septal defect and growth restriction but no brain abnormality on USS (not included in the present study). Microcephaly, agenesis of the corpus callosum (**a** coronal) with an inter-hemispheric cyst (**b** axial), and hypotelorism (**c** axial) were shown on iuMR imaging at 25 gestational weeks

**Fig. 2:**
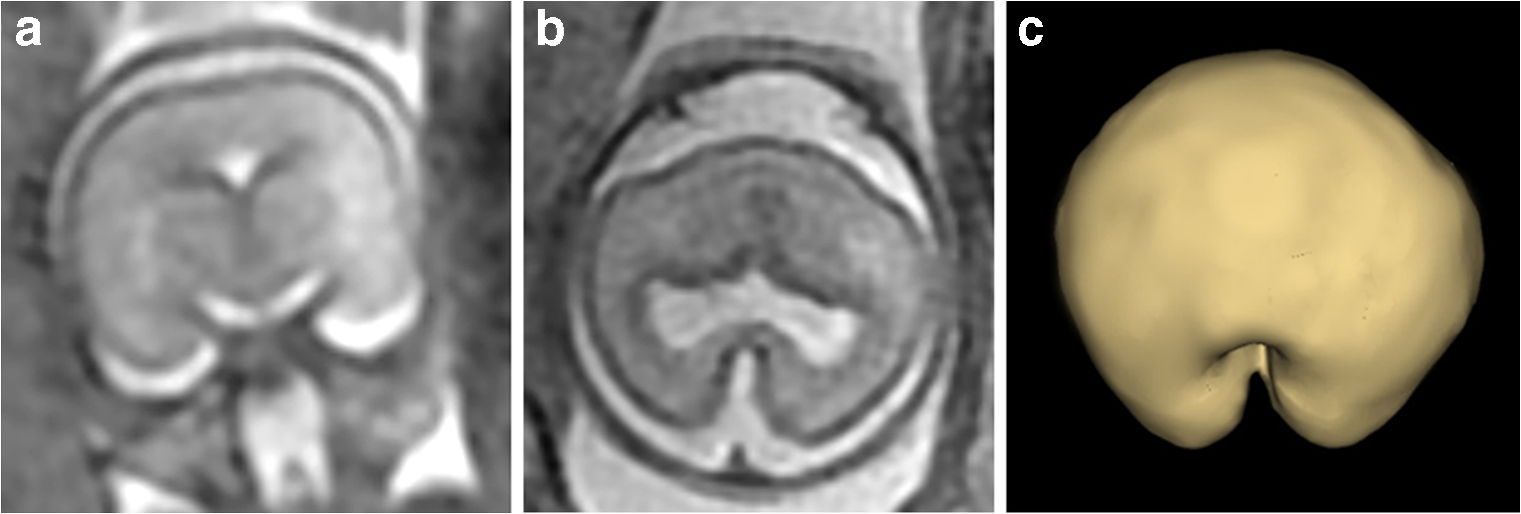
A fetus with known truncus arteriosus and “small cavum septum pellucidum” on USS (not included in the present study). Routine 2D iuMR images at 21 gestational weeks (**a** coronal and **b** axial) and a superior view of the brain surface created from 3D volume imaging (**c**) show microcephaly/micrencephaly, non-separation of the cerebral hemispheres, and hypoplasia of the frontal lobe diagnostic of semilobar holoprosencephaly

The multifactorial etiology of neuro-developmental problems in children with CHD was reviewed recently by Seed [14] who stated that there is likely to be morbidity because of undiagnosed chromosomal or monogenetic anomalies as well as peri-operative brain injury. Seed primarily discusses the significance of acquired brain injury in the fetus and child with CHD, but a recent systematic review has examined the prevalence of prenatal brain abnormalities in fetuses with CHD [15]. Three studies, with 221 cases, were suitable for inclusion in that review and reported a 28% rate of structural brain abnormalities in fetuses with CHD. This included developmental brain abnormalities, reductions in brain volume, and changes in metabolism, maturation, and brain blood flow. Two illustrative examples from our clinical practice are shown in Figs. 1 and 2.

In utero MR (iuMR) imaging is now a recognized clinical method for assessing the fetal brain, and a recent study (MERIDIAN) showed clear improvements in diagnostic accuracy and confidence for iuMR over ante-natal ultrasonography (USS), with an absolute improvement in diagnostic accuracy of > 22% [16]. It is important to note, however, that entrance criteria for MERIDIAN required a brain abnormality to be present on USS. Fetuses with CHD might be considered at “increased risk” of a brain abnormality, but the majority of USS studies will be normal; therefore, the results of MERIDIAN are not directly transferable to fetuses with CHD.

The first aim of this study was to trial an iuMR protocol that can obtain high-quality images of the fetal brain using routine ultrafast 2D sequences in order to see if this brings about any improvement in diagnostic accuracy when compared with the current clinical standard (USS). In addition, we will devise a method of obtaining volume estimates of the intracranial compartments using recently developed 3D MR methods in order to see if such measurements are feasible in a clinical setting and to look for changes in the volume of intracranial compartments in pregnancies complicated by fetal CHD.

## Methods

### Funding, ethical approval, and participants

The work reported here was supported by the NIHR-HTA by way of an extension to the main MERIDIAN study [16] and conducted under the same ethics approval (IRAS 62734) and clinical trials regulations (ISRCTN 27626961). Pregnant women who were interested in coming into the study were contacted by research staff from the Academic Unit of Radiology, University of Sheffield and were sent a patient information leaflet by email or post. A follow-up telephone call enabled queries to be answered, initial screening questions to be assessed, and eligibility for the studies confirmed. General inclusion criteria were as follows: the woman was at least 16 years old and the fetus must be a minimum of 18 gestational weeks (gw) at the time iuMR imaging was performed. Exclusion criteria consisted of inability to give informed consent, contraindications to MR imaging, or inability/unwillingness to travel to Sheffield for iuMR imaging. Written informed consent was taken on the day of the study. Participants were not paid for the study, but travel expenses were reimbursed.

### Fetuses with CHD

Fetuses with CHD were recruited from a single tertiary center (University Hospitals of Leicester) after referral from other regional centers and district general hospitals for fetal echocardiography, which was performed by experienced pediatric cardiologists with extensive experience in fetal echocardiography. All cases included in the study were first identified at 20 gw and were offered invasive procedures to check chromosomal make-up of the fetuses and had formal assessment of the brain by a fetal medicine specialist in the tertiary center. Pregnant women whose singleton fetus had a confirmed CHD (excluding “simple” cardiac defects, such as right aortic arch and isolated anomalies of venous drainage) were invited into the study provided that there was no structural brain abnormality on ante-natal USS and no known genetic or chromosomal abnormalities. The formal approach to come into the study was made following a follow-up ultrasound scan at 27–28-week gestation if they fulfilled all of the inclusion and exclusion criteria.

### Normal fetuses

We had previously created and reported a normative database of 200 women by scanning fetuses from low-risk pregnancies; no structural abnormality (brain or somatic) was recognized on ultrasonography, and no brain abnormality was seen on iuMR between 18 and 37 gw [17]. Those participants were informed about the study by way of posters and leaflets in 12/16 of the fetal medicine referral centers involved in the original MERIDIAN study [16]. These fetuses of appropriate gestational age from this group provided the normative data by which the fetuses with CHD in the current were compared.

### MR technique and image processing

All iuMR imaging studies were performed on the same 1.5-T whole body scanner (HDx, GE Healthcare, Milwaukee) using an eight-channel cardiac coil positioned over the maternal abdomen. Our standard range of clinical iuMR sequences was performed in accordance with the methods described previously [16], and these provided the basis of either confirming normality of the brain or the definition of the brain pathology. In all cases, 3D balanced, steady-state sequences (fast imaging employing steady-state acquisitions—FIESTA) were obtained in the axial plane of the fetal brain. This technique of producing 3D images of the fetal brain has been described in detail previously [18, 19]; but to summarise, the imaging parameters were TR 4 ms, TE 2 m, flip angle 60°, field of view of 320 mm × 260 mm, and an acquisition matrix of 320 × 256. A number of partitions and partition thickness were adjusted according to the size of the individual brain, with the aim of acquiring each dataset with the highest resolution possible without increasing the scan time (in the order of 20–24 s). Typical partition thickness was 1.8 to 2.6 mm with resultant voxel sizes of 0.6 × 0.5 × 1–1.3 mm after interpolation.

The 3D imaging datasets were transferred to a standard personal computer and loaded into 3D Slicer (http://www.slicer.org). Note that 3D Slicer does not have CE marking, so the results can only be used for research purposes. The brightness and contrast levels were user-selected to optimize visualisation of the CSF/brain interfaces, and the fetal brain was manually segmented. First, the ventricular compartment is segmented and the ventricular volume (VV) is calculated, followed by segmentation of the external brain surface in order to calculate the brain parenchymal volume (BPV), which excludes the CSF in the ventricles. The internal surface of the skull is segmented in order to calculate the extra-axial volume (EAV), and lastly, the three volumes are summated to derive total intracranial volume (TICV).

### Image interpretation and analysis

The iuMR studies were reviewed by an experienced pediatric neuroradiologist (PDG), and if no structural brain abnormality was identified, a letter reporting “no unexpected findings” was sent to the woman’s general practitioner. Alternatively, if a brain abnormality was detected on iuMR imaging, the findings were discussed with the relevant fetal maternal consultant and a full clinical style report was issued. Mean and standard deviation were calculated for each of the four intracranial volumes measured in the fetuses with CHD, and scatterplots were produced plotting gestational age against the intracranial volume of interest alongside the data obtained from 200 normal fetuses [17]. Multiple linear regression models were produced for each intracranial volume measured adjusting for gestational age. Only normal cases scanned between gestational weeks 28 to 36 (*n* = 95) were included in that analysis as there were no fetuses with cardiac abnormalities outside that range. *Z* scores were calculated for each volume measurement in each fetus with CHD by comparison with the mean and standard deviation values of normal fetuses of the appropriate gestational week. Z scores were also used to correlate changes in volume changes from the four intracranial compartments.

## Results

Twenty pregnant women carrying a fetus with CHD were initially recruited, but four did not attend the iuMR appointment; so, 16 fetuses with CHD were reported and are summarised in Table 1. The iuMR studies of the 16 fetuses with CHD were scanned between 28 and 36 gw, and in all cases, diagnostic quality of both 2D and 3D volume images were obtained within a 30-min table occupancy time.

**Table 1:** Summaries of the iuMR imaging findings of the brains of 16 fetuses with CHD

Case	Cardiac abnormality	Gestational age at iuMR	Structural brain abnormality on iuMR	VV (*Z* score)	BPV (*Z* score)	EAV (*Z* score)	TICV (*Z* score)
1	Transposition of the great arteries Atrioventricular septal defects	36w	No	+ 1.4	0.0	− 1.3	+ 0.7
2	Transposition of the great arteries Double outlet right ventricle	33w	Vermian hypoplasia, Mild ventriculomegaly	+ 3.0	− 0.7	− 2.1	− 1.3
3	Transposition of the great arteries Double outlet right ventricle	28w	No	− 1.1	− 1.2	− 1.1	− 1.9
4	Transposition of the great arteries	33w	No	+ 1.3	− 0.3	− 3.3	− 2.0
5	Transposition of the great arteries Double outlet right ventricle	29w	Vermian hypoplasia	+ 1.8	− 1.3	− 1.9	− 1.6
6	Tetralogy of Fallot	31w	No	− 0.7	− 2.0	− 3.2	− 3.0
7	Tetralogy of Fallot	31w	No	+ 0.9	− 4.1	− 5.0	− 5.1
8	Tetralogy of Fallot	31w	Mild ventriculomegaly	+ 1.9	− 2.8	− 3.9	− 3.7
9	Hypoplastic left heart syndrome	32w	No	− 1.0	− 1.7	− 1.4	− 1.9
10	Hypoplastic left heart syndrome	30w	No	+ 0.5	− 0.6	+ 0.3	− 0.4
11	Hypoplastic left heart syndrome	34w	Moderate ventriculomegaly	+ 7.8	− 3.0	− 1.5	− 4.0
12	Ventricular septal defect, pulmonary stenosis	32w	No	− 0.6	+ 0.4	− 0.5	− 0.1
13	Uhl’s anomaly	34w	No	+ 1.9	0.0	− 1.5	− 1.4
14	Atrioventricular septal defects	33w	No	+ 0.2	− 2.5	− 3.1	− 4.4
15	Ventricular septal defect	34w	Mild ventriculomegaly	+ 3.9	+ 0.6	+ 0.1	+ 0.9
16	Aortic arch hypoplasia	35w	No	+ 0.1	+ 1.8	− 1.9	+ 0.3

Mild ventriculomegaly is defined as trigone measurements of 10–12 mm and moderate ventriculomegaly as trigone measurements of 13–15-mm inclusive. *VV* = ventricular volume, *BPV* = brain parenchymal volume, *EAV* = extra-axial volume, *TICV* = total intracranial volume

**Fig. 3:**
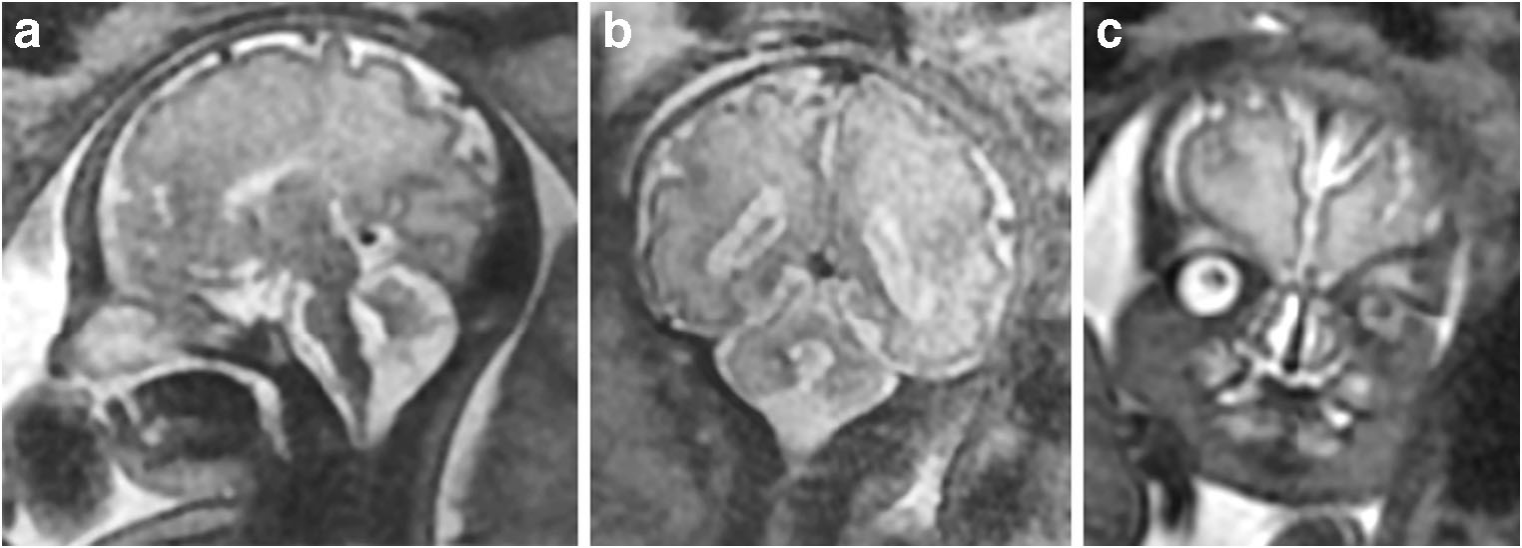
A fetus from this study (case #2 on Table 1) with known double outlet right ventricle and transposition of the great arteries but no brain abnormality on USS. Inferior vermian hypoplasia was shown on iuMR performed at 33 gestational weeks (**a** sagittal and **b** coronal) along with mild ventriculomegaly and bilateral cleft lip/palate (**a** and **c** coronal)

**Fig. 4:**
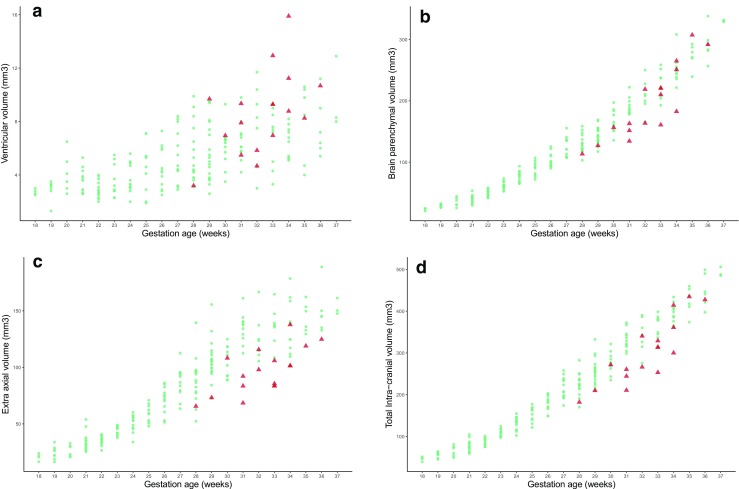
Distribution of the ventricular volume (**a** VV), brain parenchymal volume (**b** BPV), extra-axial volume (**c** EAV), and total intracranial volume (**d** TICV) for fetuses with congenital heart disease (red triangles) with the volumes measured in normal fetuses (blue circles). A summary of the statistical analysis is shown in Table 2

Intracranial structural abnormalities were recognized on 2D imaging in 5/16 (31%) fetuses with CHD and consisted of ventriculomegaly as the only intracranial structural abnormality in 3/16 and hypoplasia of the cerebellar vermis in two cases (one with ventriculomegaly as well; Table 1 and Fig. 3). In both cases, the hypoplasia of the vermis was involved the inferior portion, specifically the portion caudal to the pre-pyramidal fissure. Table 1 also shows the *Z* scores for VV, TBV, EAV, and TICV for the group of 16 fetuses with CHD when compared with the mean and standard deviations of the age-matched normal fetuses. 11/16 fetuses with CHD had TBV below the mean (i.e., negative *Z* scores), 14/16 fetuses had EAV below the mean, and 13/16 fetuses had TICV below the mean. In contrast, 12/16 fetuses with CHD had VV above the mean. Figure 4a–d shows distribution of the VV, TBV, EAV, and TICV for CHD fetuses against the normal cases, and the results of the regression models for these cases are shown in Table 2. Fetuses with CHD have statistically significant reductions in TBV, EAV, and TIBV, while VV was significantly larger when compared with controls after adjustment for gestational age.

**Table 2:** Results of multiple linear regression models on the four volume measures adjusting for gestational age

Anatomical compartment	Adjusted mean difference (95% confidence intervals)	*p* value
Ventricular volume (mm^3^)	+ 1.83 (+ 0.67, + 2.99)	0.002
Total brain volume (mm^3^)	− 19.2 (− 29.9, − 8.5)	< .001
Extra-axial volume (mm^3^)	− 30.6 (− 40.3, − 21.0)	< .001
Total intracranial volume (mm^3^)	− 48.0 (− 63.8, − 32.2)	< .001

**Fig. 5:**
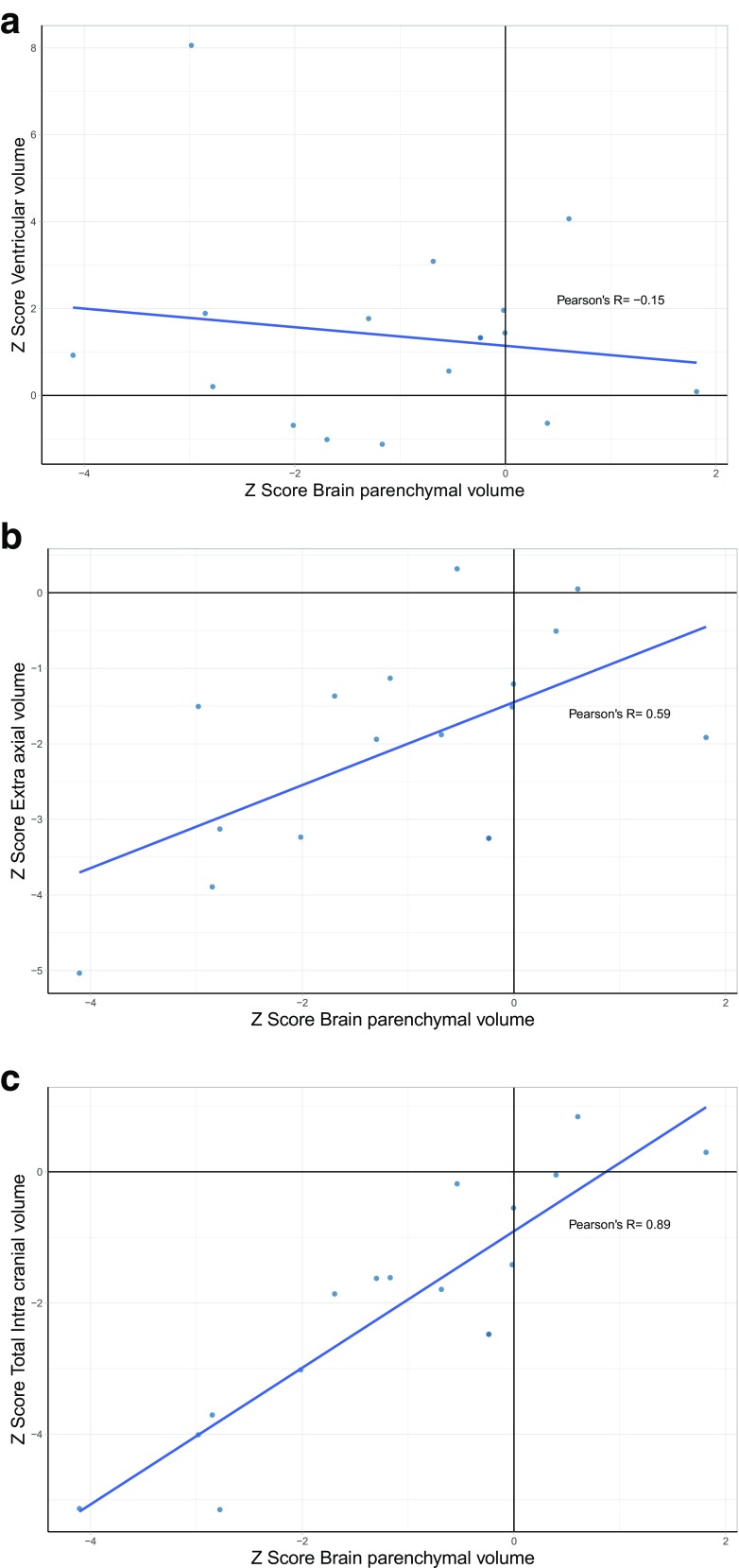
Correlation of brain parenchymal volume with ventricular volume (**a**), extra-axial volume (**b**), and total intracranial volume in 16 fetuses with CHD. See text for details

The correlation between *Z* scores for VV, EAV, and TICV compared with BPV for the fetuses with CHD is shown in scatterplots (Fig. 5a–c). Those results show that there is a strong positive correlation between TICV and BPV (Fig. 5c), a moderate positive correlation between EAV and BPV (Fig. 5b), and a very weak negative correlation between VV and BPV (Fig. 5a).

## Discussion

It has been possible to obtain high-quality ultrafast 2D images for many years, but in this paper, we have shown that it is possible to obtain high-quality, ultrafast 3D volume acquisitions of the fetal brain routinely in women with pregnancies complicated by CHD of the fetus in times that are appropriate for clinical investigations (table occupancy ≤ 30 min). Individual 2D and 3D acquisitions are obtained in 20–30 s, so that multiple attempts to obtain successful acquisitions can be made during the 30-min scan event. The most time-intensive stage of the process at present is the manual segmentation of the 3D datasets, which takes between 90 and 180 min depending on the complexity of the surface of the cortical plate/cerebral cortex.

Our prospective study showed that 5/16 (31%) of fetuses with CHD had intracranial structural abnormalities on iuMR imaging that were not detected on USS, which is similar to the 39.6% in an earlier report of Mlczoch [20] but higher than in two other reports [21, 22] as interpreted below. Two of our cases had abnormalities of the cerebellar vermis, and the other three showed varying degrees of ventriculomegaly as the only intracranial finding. We consider that all of those abnormalities are likely to be developmental in nature as there was no evidence of ischaemic injury, hemorrhage, or encephalomalacia to account for the cases of ventriculomegaly. The previously described systematic review [15] did not attempt to make a comparison of detection rates between iuMR and USS studies, but one of the studies used in the systematic review showed structural intracranial abnormalities in 23% of fetuses (33/144) with CHD with no extra-cardiac abnormalities on USS. Ten of the abnormalities consisted of enlargement of the extra-axial CSF spaces—a diagnosis that does not feature as a discrete intracranial structural abnormality in our analysis. If those cases are excluded, the abnormality rate becomes 16% (including 12 cases of mild ventriculomegaly and three fetuses with inferior vermian hypoplasia). Mlczoch et al., in contrast, did not attempt comparison between iuMR and USS findings but found intracranial abnormalities on iuMR in 39% (21/55) [20]. This group also includes nine cases of “changes of CSF spaces” that would not have been considered abnormal using the criteria used in our study, altering their results to 22%. 7/12 of the “true” brain abnormalities resulted from malformations (cortical formation abnormality (3), agenesis of the corpus callosum (2), holoprosencephaly (1), and cerebellar hypoplasia (1)), and 5/12 were thought to be acquired brain pathology (ventriculomegaly and/or ventricular bleeding (3) and germinolytic cysts (2)).

The number of studies of brain volume in fetuses with CHD reported in the systematic review of Khalil et al. [15] was small (seven studies, retrospective, not formally powered, and USS and MR methods were grouped together), but 6/7 of those described reduced brain volumes in CHD cases. Five of the studies looked at unselected forms of CHD (like our present study), four showed reduced brain volumes in CHD (three using iuMR and one using USS), and one USS-based study did not find a difference in brain volumes. The results of our study are in broad agreement with the findings of the systematic review as we have also shown statistically reduced brain volumes (i.e., reduced BPV). The major advantage of our approach is the ability to measure the other intracranial compartments as well as brain per se. This was also the approach of one study included in the systematic review, but only studied fetuses with Tetralogy of Fallot [22]. As such, we found that the fetuses with CHD had statistically smaller EAV and TICV, while their VV was increased. We also showed that there was a strong positive correlation between BPV and TICV, a moderate positive correlation between BPV and EAV, and a weak negative correlation between BPV and VV. This allows a better assessment of the possible etiological cause of the reduced BPV as different pathologies are expected to affect the intracranial compartments differently.

**Fig. 6:**
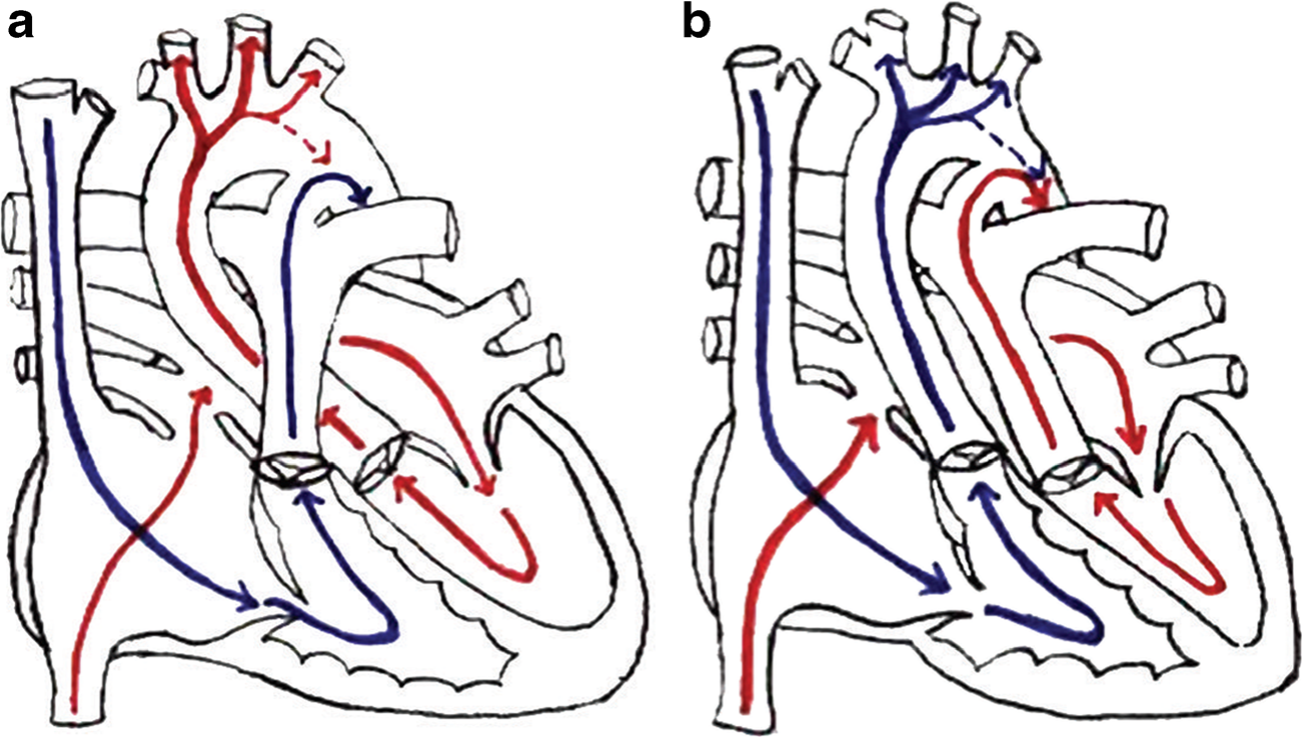
Diagrams indicating the pattern of blood flow in the normal foetal heart and in transposition of the great arteries from [7]. See text for full details but note that in the normal fetal heart (**a**), predominantly oxygenated blood (labelled red) goes into the carotid arteries (and hence the fetal brain), whereas in the fetus with transposition of the great arteries (**b**), the carotid arteries contain predominantly de-oxygenated blood (labelled blue) because the output of the right ventricle passes into the ascending aorta

Before attempting to explain the small size of brains from fetuses with CHD, it is important to review the normal circulation in the fetal heart, which is shown diagrammatically in Fig. 6a. It is a fundamental anatomical feature of the fetal heart to keep blood flow to the non-inflated lungs to a minimum, and this is achieved by shunting blood from the right atrium to the left atrium via the foramen ovale and from the pulmonary artery to the descending aorta via the ductus arteriosus. A superficial analysis of this arrangement might lead to the assumption that oxygenation levels in the blood are the same in all parts of the left side of the heart and in the major outflow tracts. However, Donofrio and Massaro [7] describe subtle, but important changes in intracardiac flow that maximize oxygen supply to the fetal brain (via the carotid and vertebral arteries) and to the fetal heart (via the coronary arteries), which are based primarily on ovine studies. Blood return to the right atrium comes from two sources, the superior and inferior vena cavae, and in the fetus, (unlike the adult) there is a marked difference in the oxygenation levels of blood in those veins. Blood in the inferior vena cava has higher levels of oxygenated blood because of the ductus venosus, which carries maternally derived oxygen via the placenta and umbilical vein. The superior vena cava, in contrast, carries high levels of deoxygenated blood from the fetal head, neck, and upper limbs. Donofrio and Massaro propose that blood from the superior vena cava (with low levels of oxygen) is directed into the right ventricle, while the eustachian valve directs oxygenated venous return from the inferior vena cava mainly into the left atrium via the foramen ovale [7]. Thus, the left side of the heart has higher levels of oxygenated blood when compared with the right side. Most of the cardiac output from the right ventricle (with low levels of oxygen) is shunted from the pulmonary arteries via the ductus arteriosus into the descending aorta where it passes to the lower part of the fetal body (the ductus arteriosus is attached distal to the origins of the carotid and vertebral arteries). So, the deoxygenated blood passes to the lower part of the fetal body and placenta, rather than to the brain. In contrast, blood with high oxygen levels in the left ventricle passes into the ascending and arch of the aorta and hence into the carotid and vertebral arteries. As a result, the normal fetal brain receives as much oxygen as possible.

The anatomically more severe forms of CHD interfere with this normal, selective passage of oxygenated blood to the fetal brain and produce intracardiac mixing with blood with lower oxygen tension. A good example occurs in transposition of the great arteries (6b) where all of the deoxygenated blood in the right ventricle is directed towards the cranio-cervical arteries and fetal brain [7]. Similar problems are encountered in fetuses with truncus arteriosus, Tetralogy of Fallot, and hypoplastic left heart syndrome [7]. Donofrio and Massaro propose that the abnormal mixing of intracardiac blood in those types CHD is associated with changes in fetal cerebral blood flow that can be detected and measured by Doppler USS. Kaltman for example showed that cerebral pulsatility index was abnormal in 7% of fetuses with CHD, all of whom had anomalies that were expected to produce abnormal cardiac mixing, which was interpreted as indicating vasodilation and increased flow in the cerebral vessels in order to compensate for specific cerebral hypoxemia [23]. We have not attempted a formal subgroup analysis examining intracranial volumes in different types of CHD because of the small number of cases in each category. We have separated the cases that are expected to produce intracardiac mixing from those that do not in Table 1, and it is clear that the severest reductions in BPV in our cohort occurred in those with expected intracardiac mixing.

Our study has several strengths. We have only included cases ≥ 28-week gestation who had a formal fetal echo assessment by pediatric cardiologists and no chromosomal or genetic abnormalities. We have excluded cases with simple ventricular septal defect, abnormal venous circulation, and or right aortic arch. We are the first group to formally assess changes in subgroup of brain volumes. The use of large number of control cases allowed formal assessment of the changes. We acknowledge the small number of cases included in our study and the inclusion of mixed major cardiac anomalies, which does not allow evaluation of each cardiac anomaly.

In summary, we describe a robust iuMR method to look at the 2D structure and measure the volumes of the different intracranial compartments of third trimester fetuses with CHD. We have shown a high rate of structural brain abnormalities that were not appreciated on USS and have shown statistically significant reductions in BPV, EAV, and TICV in those fetuses. Future large prospective adequately powered case-control studies are required to judge the clinical benefit of performing iuMR imaging in fetuses with CHD and impact on counselling. Further work is required to assess if associated structural abnormalities are directly related to the impact of cardiac anomaly or miss diagnoses. Counselling for major cardiac cases at 20-week gestation should include the possibility of late identification of brain anomalies in these cases.

## References

[1] Van der Linde D, Konings EEM, Slager MA, Witsenburg M, Helbing WA, Takkenberg JJM, Roos-Hesselink JW (2011). Birth prevalence of congenital heart disease worldwide: a systematic review and meta-analysis. J Am Coll Cardiol.

[2] Khairy P, Ionescu-Ittu R, Mackie AS, Abrahamowicz M, Pilote L, Marelli AJ (2010). Changing mortality in congenital heart disease. J Am Coll Cardiol.

[3] Majnemer A, Limperopoulos C, Shevell M, Rosenblatt B, Rohlicek C, Tchervenkov C (2006). Long-term neuromotor outcome at school entry of infants with congenital heart defects requiring open-heart surgery. J Pediatr.

[4] Limperopoulos C, Majnemer A, Shevell MI, Rosenblatt B, Rohlicek C, Tchervenkov C, Darwish HZ (2001). Functional limitations in young children with congenital heart defects after cardiac surgery. Pediatrics.

[5] Hovels-Gurich HH, Seghaye M-C, Schnitker R (2002). Long-term neurodevelopmental outcomes in school-aged children after neonatal arterial switch operation. J Thorac Cardiovasc Surg.

[6] Bellinger DC, Wypij D, DuPlessis AJ (2003). Neurodevelopmental status at eight years in children with dextrotransposition of the great arteries: the Boston Circulatory Arrest Trial. J Thorac Cardiovasc Surg.

[7] Donofrio MT, Massaro AN (2010) Impact of congenital heart disease on brain development and neurodevelopmental outcome. Int J Pediatr 359390:13. 10.1155/2010/359390 10.1155/2010/359390 PMC2938447 20862365

[8] Donofrio MT, Duplessis AJ, Limperopoulos C (2011). Impact of congenital heart disease on fetal brain development and injury. Curr Opin Pediatr.

[9] Marino BS, Lipkin PH, Newburger JW, American Heart Association Congenital Heart Defects Committee, Council on Cardiovascular Disease in the Young, Council on Cardiovascular Nursing, and Stroke Council (2012). Neurodevelopmental outcomes in children with congenital heart disease: evaluation and management: a scientific statement from the American Heart Association. Circulation.

[10] Burrows F, Hillier SC, McLeod ME, Iron KS, Taylor MJ (1990). Anterior fontanel pressure and visual evoked potentials in neonates and infants undergoing profound hypothermic circulatory arrest. Anesthesiology.

[11] Newburger J, Jonas RA, Wernovsky G (1993). A comparison of the perioperative neurologic effects of hypothermic circulatory arrest versus low-flow cardiopulmonary bypass in infant heart surgery. N Engl J Med.

[12] O’Hare B, Bissonnette B, Bohn D, Cox P, Williams W (1995). Persistent low cerebral bloodflow velocity following profound hypothermic circulatory arrest in infants. Can J Anesth.

[13] Ferry PC (1990). Neurologic sequelae of open-heart surgery in children. Am J Dis Child.

[14] Seed M (2017). In utero brain development in fetuses with congenital heart disease: another piece of the jigsaw provided by blood oxygen level-dependent magnetic resonance imaging. Circ Cardiovasc Imaging.

[15] Khalil A, Bennet S, Thilaganathan B, Paladini D, Griffiths P, Carvalho JS (2016). Prevalence of prenatal brain abnormalities in fetuses with congenital heart disease: a systematic review. Ultrasound Obstet Gynecol.

[16] Griffiths PD, Bradburn M, Campbell MJ, Cooper CL, Graham R, Jarvis D, Kilby MD, Mason G, Mooney C, Robson SC, Wailoo A (2016). Use of MRI in the diagnosis of fetal brain abnormalities in utero (MERIDIAN): a multicentre, prospective cohort study. Lancet.

[17] Jarvis DA, Finney CR, Griffiths PD (2019) Normative volume measurements of the fetal intra-cranial compartments using 3D volume in utero MR imaging. Eur Radiol. 10.1007/s00330-018-5938-5 10.1007/s00330-018-5938-5 PMC6554253 30683990

[18] Griffiths PD, Jarvis D, McQuillan H, Williams F, Paley MNJ, Armitage P (2013). 3D MR imaging of the fetal brain using a rapid steady state sequence. Br J Radiol.

[19] Jarvis DA, Armitage P, Dean A, Griffiths PD (2014). Surface reconstructions of foetal brain abnormalities using ultrafast steady state 3D acquisitions. Clin Radiol.

[20] Mlczoch E, Brugger P, Ulm B, Novak A, Frantal S, Prayer D, Salzer-Muhar U (2013). Structural congenital brain disease in congenital heart disease: results from a fetal MRI program. Eur J Paediatr Neurol.

[21] Brossard-Racine M, du Plessis AJ, Vezina G, Robertson R, Bulas D, Evangelou IE, Donofrio M, Freeman D, Limperopoulos C (2014). Prevalence and spectrum of in utero structural brain abnormalities in fetuses with complex congenital heart disease. Am J Neuroradiol.

[22] Schellen C, Ernst S, Gruber GM, Mlczoch E, Weber M, Brugger PC, Ulm B, Langs G, Salzer-Muhar U, Prayer D, Kasprian G (2015). Fetal MRI detects early alterations of brain development in Tetralogy of Fallot. Am J Obstet Gynecol.

[23] Kaltman JR, Tian Z, Rychik J (2005). Impact of congenital heart disease on cerebrovascular blood flow dynamics in the fetus. Ultrasound Obstet Gynecol.

